# Characterization of recurrent cytomegalovirus reactivations post allogenic stem cell transplantation in a population with high seropositivity

**DOI:** 10.1186/s12985-024-02421-y

**Published:** 2024-07-02

**Authors:** Hajar Y. AlQahtani, Nada AlSuhebany, Shuroug A. Alowais, Bashayer AlShehri, Abdullah Althemery, Amirah Alghanim, Hessa Alqahtani, Lama Alkhathran, Majd Alyaqub, Mariam Alsulimani, Ahmad AlHarbi, Hind Alhatmi, Sarah Almansour, Abdulellah Almohaya, Mohammed Bosaeed

**Affiliations:** 1 Department of Pharmaceutical Care, King Abdulaziz Medical City, Ministry of National Guard-Health Affairs, Riyadh, Saudi Arabia; 2 King Abdullah International Medical Research Center, Riyadh, Saudi Arabia; 3 King Saud Bin Abdulaziz University for Health Sciences, Riyadh, Saudi Arabia; 4 College of Pharmacy, King Saud Bin Abdulaziz University for Health Sciences, Riyadh, Saudi Arabia; 5 Department of Clinical Pharmacy, College of Pharmacy, Prince Sattam Bin Abdulaziz University, Al-Kharj, Al Riyadh Province Saudi Arabia; 6 Division of Infectious Diseases, Department of Medicine, King Abdulaziz Medical City, Ministry of National Guard-Health Affairs, Riyadh, Saudi Arabia; 7 College of Medicine, King Saud Bin Abdulaziz University for Health Sciences, Riyadh, Saudi Arabia

**Keywords:** Cytomegalovirus, Reactivation, Transplant, Stem cell, Allogenic

## Abstract

**Objectives:**

This study aimed to characterize incidences of CMV reactivations within one year post-allo-SCT and identify risk factors for CMV second reactivation episode in population with high seropositivity where first CMV reactivation episode deemed to be high.

**Methods:**

This retrospective cohort study analyzed data from 359 allo-SCT patients aged 14 and older admitted to a tertiary academic hospital. Data on demographic and clinical factors, CMV serostatus, conditioning regimens, graft-versus-host disease prophylaxis, engraftment time, and CMV reactivations were collected.

**Results:**

First and second CMV reactivations occurred in 88.9% and 18.4% of post-allo-SCT patients respectively. Patients were stratified into two groups based on primary disease necessitating allo-SCT, patients with malignant (Group 1) and non-malignant (Group 2) hematological disease. Factors associated with the second reactivation included cord blood as a stem cell source, human leukocyte antigen mismatch, acute graft-versus-host disease, and hematological malignancies. Patients with non-malignant hematological disease displayed better outcomes, including a higher rate of spontaneous clearance of first CMV reactivation (70% versus 49.4%) and lower rates of second CMV reactivation (9.6% versus 31%) than those with malignant hematological disease. The one-year overall survival rate was 87.7% (95.5% in non-malignant hematological disease and 78.13% in malignant hematological disease).

**Conclusion:**

Our findings are concordant with previous local study in regard to high rate of first CMV reactivation post-allo-SCT. It appears that patients with nonmalignant hematological disease had better outcomes, such as lower second CMV reactivation and higher survival rates compared to patients with malignant hematological disease. Further investigation is needed to identify other factors affecting recurrent CMV reactivations in allo-SCT in patients with malignant hematological disease.

**Supplementary Information:**

The online version contains supplementary material available at 10.1186/s12985-024-02421-y.

## Introduction

Cytomegalovirus (CMV) reactivation after allogenic hematopoietic stem cell transplantation (allo-SCT) is considered one of the main risk factors for increased mortality post-allo-SCT [1, 2]. It is associated with higher rate of graft-versus-host disease (GvHD) and bacterial and fungal infections in allo-SCT recipients [3, 4]. The global average prevalence of CMV seropositivity was estimated to be as low as 66% in European regions which considered to be the lowest and as high as 90% in Eastern Mediterranean which considered to be the highest [5]. CMV seropositive status is one of the important predicting factors for post-allo-SCT CMV reactivation owing to its ability to regulate immune system response [6].

Onset of CMV reactivation is typically reported within 28 to 72 days after transplantation; affecting 40–60% of bone marrow allogeneic transplant recipients and may progress to severe CMV-related diseases [7–9]. It is worth mentioning that variation in CMV reactivation definition among clinical studies may contribute to the wide ranges of CMV reactivation rates seen in literature. Two strategies are presented to prevent CMV reactivation, prophylaxis and preemptive therapy. Preemptive therapy used to be the standard of care to avoid toxicities associated with antiviral therapy; until letermovir approved in 2017 [6]. Antiviral drugs are valganciclovir, ganciclovir and foscarnet that are well known for their clinical effectiveness in reducing CMV viral load and their toxicities (hematological toxicity with valganciclovir and ganciclovir; nephrotoxicity with foscarnet) [6, 10, 11]. The American Society for Transplantation and Cellular Therapy (ASTCT) recommended letermovir prophylaxis for adult CMV seropositive allogeneic SCT recipients, to begin no later than 28 days after SCT and continuing through day 100 [12].

Letermovir is an antiviral agent that inhibits the CMV DNA terminase complex, a requisite for viral DNA processing and packaging. It received approval from the Food and Drug Administration in 2017 for prophylaxis in adult seropositive SCT recipients [13]; notably, this agent was more effective in lowering the incidence of CMV reactivation than placebo without compromising engraftment in CMV-seropositive patients [13]. To date, letermovir is a non-formulary drug in most healthcare institutions in Saudi Arabia, including our institution the Ministry of National Guard Health Affairs (MNGHA).

A previous study conducted by *Damlaj* et al. at the MNGHA has described CMV reactivation after allo-SCT in 195 patients and revealed that over 90% of patients developed first episode of CMV reactivation post-allo-SCT [14]. CMV reactivation prevention strategy followed at our institution is preemptive therapy. The cutoff CMV viral load that triggers starting antiviral therapy is more than or equal to 1000 copies/mL. Additionally, they identified several risk factors associated with decreased overall survival at two years, including advanced age, underlying malignant diseases, and chronic graft-versus-host disease (cGvHD).

Evidently, data describing risk factors associated with recurrent CMV reactivations in populations with high rate of first CMV reactivation post-allo-SCT are lacking. Hence, this study aimed to identify the incidence of first and second recurrent CMV reactivations post-allo-SCT and risk factors for second recurrent CMV reactivation episode considering the high rate of first recurrent CMV reactivation post-allo-SCT.

## Materials and methods

### Study design and setting

This was a retrospective single-center cohort study of patients who underwent allo-SCT between April 2014 and June 2022. Patients aged 14 years or older were required to have undergone serial CMV quantitative PCR post-allo-SCT, monitored at least once weekly during the initial 90 days post-transplantation, and then bi-weekly thereafter, to be included in the study. Number of patients with no CMV reactivation was recorded to identify rate of first CMV reactivation but excluded from analysis. Patients who died within 30 days of transplantation with no documented CMV reactivation episodes and those who received antiviral treatment for CMV DNAemia without CMV quantitative PCR testing for monitoring were excluded from the study.

This study received Institutional Review Board approval (NRC22R/011/01) from the King Abdullah International Research Center at the MNGHA Hospital in Riyadh, Saudi Arabia.

### Data collection and statistical analysis

The following data were collected: demographic and baseline comorbidities, underlying disease, conditioning regimen, source of stem cell transplant, type of donor (human leukocyte antigen [HLA] typing), type of GvHD prophylaxis if received, time for engraftment, presence of acute and/or chronic GvHD, CMV donor-recipient serostatus, time to first CMV reactivation after transplantation, and need for antiviral therapy for CMV DNAemia with peak levels of serum CMV PCR. Non-relapse mortality at 90 days post-allo-SCT and overall one-year survival were also collected. Furthermore, data were analyzed based on allo-SCT recipients with malignant and non-malignant hematological diseases. First and second CMV reactivation post-allo-SCT and spontaneous clearance were monitored and recorded for both groups in this study.

The main outcome variables were the incidence of first and second CMV reactivations within one year of clearing the first episode of CMV reactivation as the primary outcomes. Non-relapse mortality 90 days after stem cell transplantation (SCT) and overall one-year survival were the secondary outcomes.

The main independent variable was the hematological malignancy status, which was classified into two groups: yes and no. Other control variables included age, sex, body mass index (BMI), liver disease, diabetes mellitus, heart failure with reduced ejection fraction, hypertension, renal disease, stroke, bedridden status, smoking, respiratory disease, conditional regimen, source of stem cell transplant, type of donor, donor-recipient CMV serostatus, receiving GvHD prophylaxis including calcineurin inhibitors, methotrexate, mycophenolate, cyclophosphamide, systemic steroid, acute GvHD, chronic GvHD, grade I-IV of acute GvHD, viral load at first reactivation, and peak viral level before starting anti-CMV if indicated.

A series of univariate tests, including chi-square and Student’s t-tests, were used to compare patients with and without hematological malignancies, with a significance level of *p* < 0.05. The data were analyzed using R Core Team (2020).

Three independent logistic regression models were generated to predict the main dependent variables (second CMV reactivation, non-relapse mortality at 90 days post-allo-SCT and overall one-year survival). The models were based on an automatic stepwise selection process from the list of independent and control variables. All intended variables were entered into the model at a significance level of 0.3 to enter the model, and a significance level of 0.35 was required for a variable to stay in the model.

### Definitions

- *CMV monitoring*: Whole blood specimen was collected in EDTA tubes then centrifuged for which plasma (supernatant) was transferred to another tube. QIAGEN EZ1 assay was used for Viral DNA extraction and Abbott Real Time CMV m2000rt assay for CMV amplification following standardization reference according to the 1^st ^WHO international standard techniques. The sensitivity of the assay is 20 copies/mL (31.2 IU/mL) of plasma. Abbott Real Time CMV assay targets UL34 and UL80.5 genes sequence within CMV genome which are highly specific and conserved for CMV. Rare mutations might be present in highly conserved regions of CMV genome for which Abbott Real Time CMV assay mitigate this risk by amplifying two selected targets of the conserved regions as indicated in the product package insert [15].
- *First recurrent CMV reactivation *post-allo-SCT was defined as the first detectable CMV viral load from blood samples post-allo-SCT (viral load > 20 copies/mL) for at least two consecutive tests, one week apart [16].
- *Second recurrent CMV reactivation *post-allo-SCT was defined as the first detectable CMV viral load from blood samples post-allo-SCT (viral load > 20 copies/mL) within one year (must be > 4 weeks) after clearing the first CMV reactivation for at least two consecutive tests, one week apart [16].
- *Spontaneous clearance *of CMV DNAemia was defined as the first two negative CMV PCR tests from blood samples one week apart, without any medical intervention [16].
- *CMV DNAemia clearance* was defined as a spontaneous clearance but with medical intervention (anti-CMV therapy).
- *Conditioning regimen*

*Myeloablative conditioning *(MAC) was defined as high-dose of chemotherapy (alkylating agent) with or without total body irradiation based regimens [17]. It does not allow for autologous hematological recovery secondary to complete ablation of bone marrow [18].

*Reduced intensity conditioning *(RIC) was defined based on the Center for International Blood and Marrow Transplant Research (CIBMTR) operational definitions discussed at Reduced-Intensity Conditioning Regimen Workshop (illustrated below) [19].

- TBI less than or equal to 500 cGy as a single fraction or less than or equal to 800 cGy if fractionated.
- Total busulfan less than or equal to 9 mg/kg of oral busulfan
- Total melphalan less than or equal to140 mg/m [2].
- Thiotepa less than 10 mg/kg

*Non-myeloablative *(NMA) conditioning was defined by regimens that resulted in minimal cytopenia with no stem cell support needed [17].

- *Engraftment* was defined as the first of three consecutive days with an absolute neutrophil count higher than 0.5 × 10^9^/L (sustained > 20 × 10^9^/L platelets and hemoglobin > 80 g/L and free of transfusion requirements).

## Results

### Demographic characteristics

**Fig. 1:**
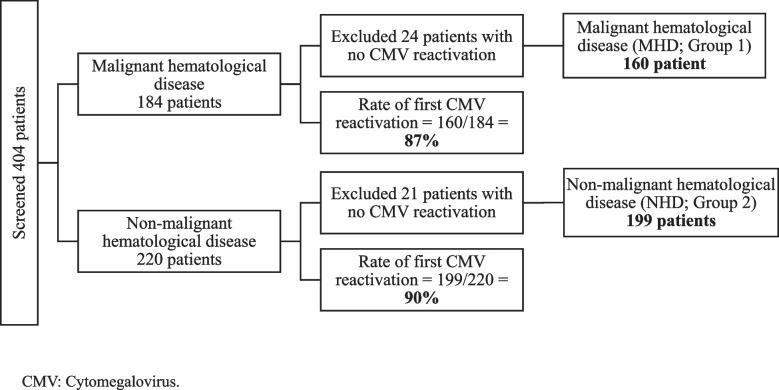
Flowchart illustrating patient screening and inclusion and exclusion criteria. CMV: Cytomegalovirus

A total of 404 patients were screened between April 2014 and June 2022, 359 of whom met the inclusion criteria (Fig. 1). Of the excluded patients, 45 were excluded because of the absence of CMV first reactivation post-allo-SCT; hence, the incidence of patients with documented first CMV reactivation post-allo-SCT from the total screened patients was 88.9% (*n* = 359/404). The patients were subsequently categorized into two groups based on the underlying disease necessitating allo-SCT: patients with malignant hematological diseases (MHD; Group 1, *n* = 160) and those with non-malignant hematological diseases (NHD; Group 2, *n* = 199).

Table 1 displays the characteristics of patients who experienced documented first CMV reactivation post-allo-SCT. These patients were predominantly male (57%), had an average age of 29 years, and an average BMI of 24 kg/m^2^.

**Table 1:** Baseline characteristics of allogenic SCT with CMV reactivations

Variable	Malignant Hematological Disease (MHD) Group 1 *N* = 160	Non-malignant Hematological Diseases (NHD) Group 2 *N* = 199	Total *N* = 359	*p* value
**Demographics**				
Age, in years, mean ± S.D	33.87 ± 14.2	25.49 ± 7.4	29.22 ± 11.7	0.001
Male, N (%)	95 (59.4)	110 (55.3)	205 (57.1)	0.44
BMI, in Kg/m^2^, mean ± S.D	25.7 ± 6.1	23.7 ± 6.2	24.4 ± 6.2	0.01
**Baseline comorbidities, N (%)**				
Cirrhosis /liver disease	4 (2.5)	6 (3)	10 (2.8)	0.24
DM	22 (13.8)	12 (6)	34 (9.5)	0.01
HFrEF < 45%	6 (3.8)	11 (5.5)	17 (4.8)	0.43
HTN	11 (6.9)	8 (4)	19 (5.3)	0.23
Renal disease (eGFR < 30%)	6 (3.8)	12 (6)	18 (5)	0.33
Stroke/ neurological disease	4 (2.5)	28 (7.8)	32 (8.9)	0.001
**Underlying Diseases, N (%)**				
Acute lymphoblastic leukemia	62 (38.8)	0	62 (17.3)	
Acute myeloid leukemia	62 (38.8)	0	62 (17.3)	
Hodgkin lymphoma	10 (6.3)	0	10 (2.8)	
Non-Hodgkin lymphoma	8 (5)	0	8 (2.2)	
Chronic myeloid leukemia	6 (3.4)	0	6 (1.7)	
Myelodysplastic syndrome	6 (3.4)	0	6 (1.7)	
Multiple Myeloma	6 (3.4)	0	6 (1.7)	
Sickle Cell anemia	0	180 (90.5)	180 (5)	
Aplastic anemia	0	16 (8)	16 (4.5)	
Others^b^	0	3 (1.5)	1 (0.8)	
**Bone Marrow Transplantation Therapy**				
Conditioning regimen, N (%)				0.001
Myeloablative	121 (75.6)	6 (3)	127 (35.4)	
Reduced intensity (RIC)	33 (20.6)	28 (14)	61 (17)	
Non-myeloablative	6 (3.8)	165 (82.9)	171 (47.6)	
Source of stem cell, N (%)				0.003
Peripheral blood stem cell	157 (98.1)	180 (90.5)	337 (93.9)	
Cord blood	3 (1.9)	19 (9.6)	22 (6.1)	
Type of donor, N (%)				0.001
HLA-matched related	118 (73.8)	182 (91.5)	300 (83.6)	
HLA-matched unrelated	16 (10)	5 (2.5)	21 (5.9)	
HLA-mismatched	26 (16.3)	12 (6)	38 (10.6)	
Donor-recipient CMV serostatus, N (%)				0.14
D^+^/ R^+^	150 (93.8)	174 (87.4)	324 (90.3)	
D^**−**^/ R^+^	3 (1.9)	13 (6.5)	16 (4.5)	
D^+^/ R^**−**^	6 (3.8)	9 (4.5)	15 (4.2)	
D^**−**^/ R^**−**^	1 (0.6(	3 (1.5)	4 (1.1)	
Engraftment, N (%)^a^				0.09
Within 7 days	28 (17.5)	30 (15.1)	58 (16.2)	
Within 8–14 days	14 (8.7)	16 (8)	30 (8.4)	
Within 15–21 days	63 (39.4)	82 (41.2)	145 (40.1)	
Within 22–28 days	13 (8)	40 (20.1)	53 (14.8)	
> 28 days	10 (6.3)	17 (8.5)	27 (7.5)	

*BMI* Body mass index, *SD* Standard deviation, *HLA* Human leukocyte antigen, *eGFR* estimated glomerular filtration rate, *DM* Diabetes Mellitus, *HFrEF* Heart Failure with Reduced Ejection Fraction, *HTN* Hypertension, *CMV* Cytomegalovirus, *SCT* Stem cell transplantation

^a^Data are missing for 46 patients

^b^Others: Thalassemia, paroxysmal nocturnal hemoglobinuria and Langerhans cell histiocytosis

### Malignant hematological diseases (Group 1)

In the MHD group, one hundred and sixty patients had a mean age of 34 years, and approximately 60% of them were male. Only 13% had at least one comorbidity, with diabetes mellitus being the most common, followed by hypertension. The most common underlying diseases necessitating allo-SCT were acute lymphoblastic leukemia and acute myeloid leukemia in approximately 75% of patients. Furthermore, more than 75% of the patients received a myeloablative regimen during the conditioning phase. The source of stem cells was primarily peripheral blood (> 90%). More than 80% of stem cell recipients received HLA-matched donors, whereas only 16% received mismatched donors. Notably, approximately 94% (~ 150) of patients had a D + /R + CMV serostatus. Importantly, nearly half of the patients developed acute GvHD (33.75%; 54/160) that progressed to chronic GvHD (48%; 26/54).

Overall, majority of patients had acute lymphoblastic leukemia, received myeloablative regimen for conditioning regimen and received HLA- matched donor. Supplementary data are presented in Table 1.

### Non-malignant hematological diseases (Group 2)

A total of 199 patients with an average age of 26 years were predominantly male (55%), with a BMI of 23.7 kg/m^2^. Stroke and other neurological diseases, followed by diabetes mellitus and renal disease were the most frequently observed comorbidities.

More than 90% of the patients in this group had sickle cell anemia and aplastic anemia as the underlying diseases necessitating allo-SCT. Most patients received a non-myeloablative conditioning regimen, followed by a reduced-intensity regimen (83% and 14%, respectively). Similar to Group 1, the source of stem cells was peripheral cells in > 90% of the patients. Most of the patients had an HLA-matched donor. Moreover, the CMV serostatus was positive for donors and recipients in more than 84% of the patients, followed by negative donors and positive recipients. Most patients achieved full engraftment at 3 weeks post-SCT. Finally, 21 patients developed acute GvHD, with most patients having Grade I GVHD (57%; *n* = 12).

Predominantly, patients had sickle cell anemia and received non-myeloablative conditioning regimen. Rate of acute GvHD is significantly lower compared to Group1 (10.55% versus 33.75%, *p* = 0.001). Further details are provided in Table 1 and supplementary Table 1.

### Incidence of recurrent CMV reactivations post-allo-SCT

#### Recurrent CMV reactivations in the group 1:

The mean time between allo-SCT and the first episode of recurrent CMV reactivation was 23.8 days, with an average first-detected viral load of 3670 copies/mL. The percentage of patients who spontaneously cleared CMV DNAemia without needing anti-CMV treatment was 49.4% (*n* = 79) at a mean time of 55 days. Twenty-nine percent of the patients (*n* = 47) were admitted for febrile neutropenia, with confirmed bacterial infections in approximately 90% of them. Thirty-eight percent of patients received antibiotics for either febrile neutropenia and/or bacterial infection, with the most frequently prescribed antibiotics being meropenem, vancomycin, and piperacillin/tazobactam. Patients developed DNAemia and necessitated anti-CMV therapy, specifically with valganciclovir, ganciclovir, and foscarnet, for 48, 46, and 13 patients, respectively. A total of 79 patients (49.4%) received anti-CMV treatment, among whom 67 (85%) successfully cleared CMV viremia through treatment. Moreover, seven patients developed CMV disease, manifesting as pneumonitis (*n* = 4), colitis (*n* = 2), and hepatitis (*n* = 1). Further details are available in Table 2.

**Table 2:** Description of first and second recurrent CMV reactivations, CMV pre-emptive therapy and disease

Variable	Malignant Hematological Disease (MHD) Group 1 *N* = 160	Non-malignant Hematological Diseases (NHD) Group 2 *N* = 199	Total *N* = 359	*p* value
**First CMV recurrent reactivation post allo-SCT (*N* = 359)**				
Time from transplant until first reactivation, days, mean ± SD	23.8 ± 29.3	16.6 ± 13.5	19.79 ± 22.2	0.004
Viral load at first reactivation, copies/mL, mean ± SD	3670 ± 44,552	105 ± 217	1706 ± 29,745	0.3
Viral peak level, copies/mL, mean ± SD	12,206 ± 54,797	5537 ± 42,754.6	8509 ± 48,534	0.21
Spontaneous clearance of CMV DNAemia, N (%)	79 (49.4)	139 (70)	218 (61)	0.001
Time to spontaneous clearance in days, mean ± SD	55 ± 41	57 ± 36	56 ± 38	0.70
Anti-CMV therapy for pts who did not achieve spontaneous clearance after first episode, N (%)	79 (49.4)	60 (30)	139 (38.7)	0.001
Anti-CMV prescribed, N (%)				
Valganciclovir	48 (30)	33 (16.6)	81 (22.6)	0.001
Ganciclovir	46 (28.8)	25 (12.6)	71 (19.8)	0.001
Foscarnet	13 (8.13)	21 (10.6)	34 (9.5)	0.4
CMV DNAemia cleared after anti CMV therapy, N (%)^a^	67 (85)	53 (88)	120 (86.3)	0.15
CMV disease	7 (4.4)	2 (1)	9 (2.5)	0.08
**Second CMV recurrent reactivation post allo-SCT (*N* = 349)**				
Incidence of second recurrent CMV reactivation, N (%)^c^	47 (31)	19 (9.6)	66 (18.9)	0.03
Time from clearance of first CMV reactivation episode to second reactivation, days, mean ± SD	82 ± 67	44 ± 25	71 ± 60	0.002
Viral load at first reactivation, copies/mL, mean ± SD	329 ± 1732	79 ± 116	257 ± 1468	0.2
Viral peak level, copies/mL, mean ± SD	3530 ± 13,659	2364 ± 8196	3194 ± 12,348	0.3
Spontaneous clearance of CMV DNAemia, N (%)^b^	35 (74.5)	16 (84.2)	51 (77.3)	0.04
Time to spontaneous clearance in days, mean ± SD	49 ± 26	54 ± 39	51 ± 30	0.1
Anti-CMV therapy for pts who did not achieve spontaneous clearance after second episode, N (%)	12 (25.5)^d^	3 (15.8)^e^	15 (22.7)	0.06

*CMV* Cytomegalovirus, *SD* Standard Deviation

^a^*N* = 139

^b^*N* = 66

^c^Excluded patients who died before second reactivation (8 patients in Group1 and 2 patients in Group 2)

^d^6 patients died and 6 patients cleared CMV DNAemia

^e^3 patients died

**Fig. 2:**
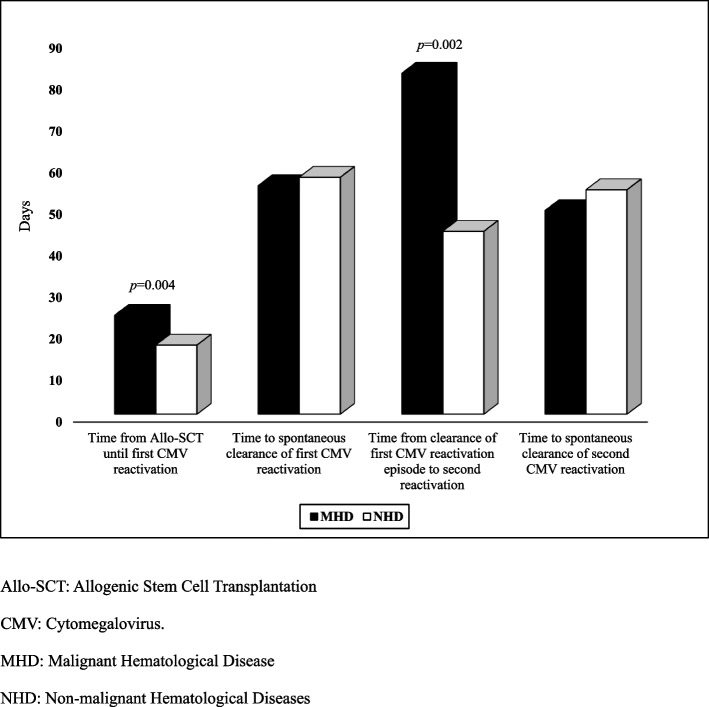
Onset of recurrent CMV reactivations and spontaneous clearance. Allo-SCT: Allogenic Stem Cell Transplantation, CMV: Cytomegalovirus. MHD: Malignant Hematological Disease, NHD: Non-malignant Hematological Diseases

**Fig. 3:**
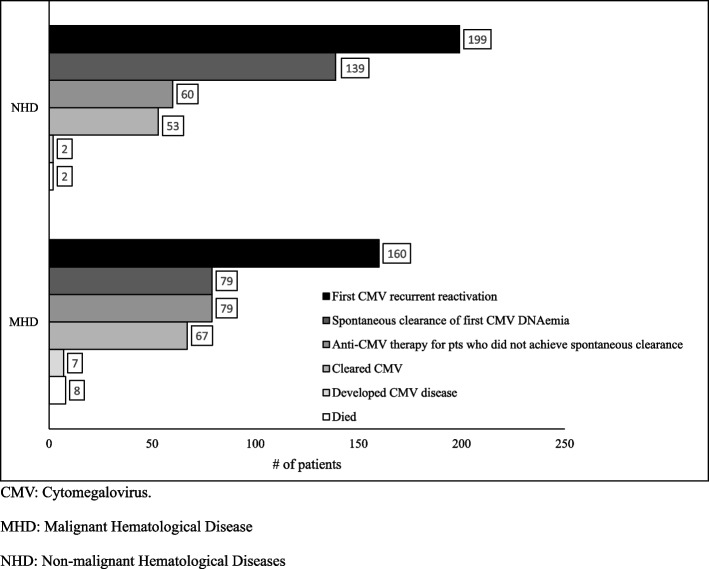
Disposition of patients with first CMV recurrent reactivation. CMV: Cytomegalovirus. MHD: Malignant Hematological Disease. NHD: Non-malignant Hematological Diseases

Thirty one percent of patients developed second recurrent CMV reactivation within an average time of 82 days post clearance of first episode of CMV reactivation post-all-SCT. Viral load levels and rate of spontaneous clearance improved compared to the first CMV recurrent reactivation episode. More details in Table 2 and Figs. 2 and 3

#### Recurrent CMV reactivation in the group 2:

The average time for first recurrent CMV reactivation was approximately 17 days post-allo-SCT, with an average viral load of 107 copies/mL at the first documented activation. The viral load peaked at an average of 5537 copies/mL. Notably, seventy percent of patients (70%, *n* = 139/199) experienced spontaneous clearance within an average time of 57 days following reactivation. Approximately 25% of the patients with first recurrent CMV reactivation developed febrile neutropenia, and 18% had confirmed bacterial infections. Those who did not achieve spontaneous clearance were treated with anti-CMV therapy (30%, *n* = 60/199), utilizing valganciclovir (*n* = 33), ganciclovir (*n* = 25), or foscarnet (*n* = 21). Of these, 88% achieved CMV DNAemia clearance (*n* = 53/60), and within this subgroup, two patients developed CMV diseases. Further details are provided in Table 2.

**Fig. 4:**
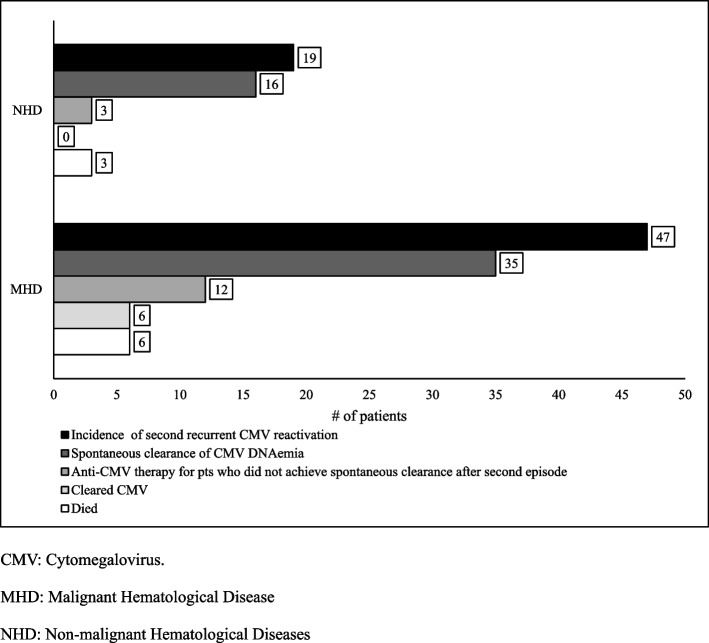
Disposition of patients with second CMV recurrent reactivation. CMV: Cytomegalovirus. MHD: Malignant Hematological Disease. NHD: Non-malignant Hematological Diseases

Approximately, 10% of patients developed second recurrent CMV reactivation within 44 days after the clearance of first episode. Similarly, second episode of CMV reactivation in this group was associated with better outcomes in terms of viral load levels and rate of spontaneous clearance as in MHD group. Full descriptive data provided in Table 2 and Figs. 2 and 4

### Non-relapse mortality at 90 days post-allo-SCT and one-year overall survival rate

The non-relapse mortality at 90 days post-allo-SCT was 3.8%, with one-year overall survival rate of 78.13% in MHD group. In contrast, in the NHD group, three patients died within 90 days after allo-SCT and the one-year overall survival was 95.5%.

The one-year overall survival in the MHD group was significantly lower than did those in the NHD group (78.13% vs. 95.5%, *p* < 0.001).

### Predictors of second recurrent CMV reactivation risk and overall survival at one year

Factors associated with an increased rate of second recurrent CMV reactivation within one year following the initial episode of first recurrent CMV reactivation post-allo-SCT include cord blood as a source of transplanted stem cells, HLA mismatch, patients with acute GvHD, and those with hematological malignancies; the respective Odds Ratios and 95% Confidence Intervals, along with *p*-values, are as follows: (2.7; 1.05–6.88, *p* = 0.04), (6.8; 2.44–19.31, *p* = 0.0008), (2.1; 1.01–4.36, *p* = 0.047), and (2.98; 1.55–5.72, *p* = 0.001).

For first-year overall survival, patients who received cord blood as a source of SCT had a higher risk for death at 1 year than did those who did not (Odds Ratio [OD]; 95% Confidence Interval [CI], *p*-value: 0.20; 0.08–0.538, 0.001). Similarly, those with hematological malignancies had a higher risk for death at 1 year compared than did those without such malignancies (OD; 95% CI, *p*-value: 0.223; 0.098–0.506, 0.0003) (Table 3).

**Table 3:** Multivariate logistic regression analysis of predictors and risk factors for second CMV reactivation within one-year after clearing first episode of CMV reactivation post allo-SCT and overall survival in one year

*Factors*	*Odd ratios (C.I)*	*p value*
**Predictors for second recurrent CMV reactivation**		
> *Donor source (umbilical cord cell)*	2.7 (1.059–6.887)	0.04
> *Type of donor ( HLA-mismatched)*	6.874 (2.446–19.319)	0.0008
> *Acute GvHD*	2.101 (1.011–4.366)	0.047
> *Hematological malignancies*	2.987 (1.558- 5.726)	0.001
**Predictors for overall survival in one year**		
> *Donor source (umbilical cord cell)*	0.207 (0.080–0.538)	0.0012
> *Hematological malignancies*	0.223 (0.098–0.506)	0.0003

*CMV* Cytomegalovirus, *GvHD* Graft-versus-Host Diseases

Controlling for age and presence of diabetes mellitus

Only statistically significant results were included in this table

## Discussion

This study was the first to evaluate the first and second recurrent CMV reactivation episodes in patients who underwent allo-SCT in a population with high CMV seropositivity; stratified based on underlying malignant and non-malignant hematological diseases. Unsurprisingly, 89% of patients experienced first recurrent CMV reactivation, regardless of their underlying disease; however, the outcomes were different [14]. Outcomes such as the rate of CMV DNAemia spontaneous clearance, second recurrent CMV reactivation, and overall survival at 1 year were all favorable in the NHD group compared to those in the MHD group. Specifically, the second recurrent CMV reactivation, patients with hematological malignancies were at a higher risk of the second recurrent CMV reactivation (31%) than those with non-malignant hematological diseases (9.6%). Compared with other study, the overall second reactivation rate was (24%), with the majority of patients having hematological malignancies and CMV D + /R + or D-/R + serology in > 80% of the patients [20]. Moreover, only (65%) of patients experienced first CMV reactivation post-allo-SCT. Authors utilized preemptive approach for prevention of CMV reactivation where CMV viral load threshold was not described in their study. Additionally, CMV reactivation was defined as CMV viral load necessitating anti-CMV therapy which might explain the differences between our results and theirs. Thus, direct, and meaningful comparison between two studies is impossible taking into consideration previously mentioned limitations.

In the NHD group of our cohort, we made a comparison with the results of the study by *Takenaka* et al., who investigated CMV reactivation in patients with aplastic anemia undergoing allo-SCT. The rates of first recurrent CMV reactivation were 90% and 55% in our and *Takenaka's* cohorts, respectively, which can be attributed to the notably high rate of seropositivity in our cohort and different definition of CMV reactivation in their study (CMV viremia > 500 copies/mL). *Takenaka* et al. calculated the overall survival for a period of 3 years post-allo-SCT, making it challenging to directly compare with our 1-year overall survival rates (88% versus 95.5%) [21]. It is conceivable that the overall survival could decline in our cohort if monitoring were extended over a longer period.

A higher proportion of patients in the NHD group were able to spontaneously clear CMV DNAemia, exhibited lower average CMV viral peak levels, and manifested a reduced rate of second recurrent CMV reactivation (9.6%). Contributing factors to this reduced rate include a smaller proportion of patients receiving cord blood as a source of transplanted stem cells and fewer instances of mismatched-related and acute GvHD. In contrast, the MHD group demonstrated divergent behavior concerning the aforementioned outcomes, making it evident that hematological malignancy triples the risk of second recurrent CMV reactivation. The lack of comparative studies for assessing risk factors associated with second recurrent CMV reactivation posed challenges in the preparation of this manuscript. However, this appears to be the first study to address this issue within an allo-SCT population. Moreover, *Wagner-Drouet* et al. reported the rates of recurrent CMV reactivations for the first and second episodes without evaluating risk factors (*n* = 101/154, 65.6%; 24/101, 19.8%) [20].

In our study population, we observed a positive association between acute GvHD and second recurrent CMV reactivation. This finding is consistent with previous studies that proven this association with first CMV reactivation [22–25]. The results of the study by *Akahoshi* et al., who examined the development of grade II-IV acute GvHD in patients with first CMV reactivation, showed bidirectional relationship between both [25].

In multivariate analysis, HLA mismatch was associated with a six-fold increase in the incidence of second recurrent CMV reactivation; however, it could be augmented by the presence of other factors, such as acute GvHD and umbilical cord cells as a source of transplanted stem cells and type of GvHD prophylaxis received [25–27].

High-dose steroids were not significantly associated with a greater risk of CMV second reactivation in our population, contradicting the results of a recent study by *Suarez-Liedo* et al. [28]. They found a significantly higher incidence of CMV reactivation and recurrence in the group with D + /R + CMV serostatus who received steroids than in patients who did not receive steroids in the same group [28]. Moreover, the effects of high-dose steroids on CMV-specific immune reconstitution remain controversial [29]. Some studies have shown that patients with allo-SCT who receive high-dose steroids have lower levels of CD8 + /INF + lymphocytes (cytomegalovirus-specific CD8 + T cells) than those who do not receive high-dose steroids do [28, 30], whereas others showed steroid use is associated with qualitative rather than quantitative dysfunctional CMV- CD8 + T cells [31].

It is notable that time for CMV reactivations were significantly longer in patients with hematological malignancies compared to those with non-malignant diseases as illustrated in Fig. 2. One explanation would be that CMV reactivations might initially happen in the peripheral organs/ tissues and could not achieve detectable amount of CMV DNAemia in the blood of patients with hematological disease in a timely fashion [32]. This opens the door to discuss the utility of CMV- specific CD8 + T cells for diagnosis of CMV reactivation in situations where CMV DNA plasma level test result is unsatisfactory.

Letermovir is indicated for CMV prophylaxis in CMV-seropositive allo-SCT patients starting between days 0 and 28 post-SCT and extended to day 100 for CMV prophylaxis [8]; if unavailable owing to cost, access, or other factors, a preemptive approach is recommended [19]. In a pivotal phase 3 randomized clinical trial, compared to placebo, letermovir significantly reduced the incidence of clinically significant CMV infection through week 24 post-allo-SCT (18.9% vs. 44.3%; *p*< 0.001) [8]. This significant finding led to its approval as primary prophylaxis against CMV in CMV-seropositive allo-SCT patients. Another study in which letermovir was administered to high-risk patients indicated that letermovir effectively prevented and delayed CMV infections [20]. The results revealed a significant reduction in CMV infection rates compared to data from the period before the introduction of letermovir (10% vs. 54%, *p* < 0.001). However, several healthcare institutions in Saudi Arabia have yet to integrate letermovir as a standard policy; instead, it is often used as a non-formulary alternative, primarily for secondary CMV prophylaxis. Based on our study findings, which highlight a significant prevalence of CMV-seropositive patients and, consequently, a high occurrence of CMV reactivations, we advocate the inclusion of letermovir in the formulary and its integration as a standard policy for allo-SCT infection prophylaxis in our institution to mitigate CMV-related risks and complications. Lastly, the presence of hematological malignancies tripled the occurrence risk of second recurrent CMV reactivation and impacted overall survival at one year, with a 20% probability of decreased survival by one year.

Nonetheless, this study had several limitations. First, this was a single-center, retrospective study. Furthermore, owing to the retrospective nature of this study, some missing data could bias the results as possible confounding factors that could hinder the statistical analysis. Therefore, future prospective multicenter studies are needed to identify additional risk factors for CMV reactivations.

## Conclusion

Our study showed that majority of allo-SCT patients experienced first recurrent CMV reactivation, possibly because of the high prevalence of CMV seropositivity before transplantation. Other outcomes, such as the rate of spontaneous clearance of CMV DNAemia, second CMV reactivation, and overall survival at one year, were all favorable in the NHD group compared with those of the MHD group which highlights the significance impact of underlying disease on recurrent CMV reactivations.

### Supplementary Information

Supplementary Material 1.

## Data Availability

Data and materials involved in this study are available upon request from the corresponding author.
