# Supplementary material for: Characterization of recurrent cytomegalovirus reactivations post allogenic stem cell transplantation in a population with high seropositivity
Source: Virol J. 2024 Jul 2;21:149. doi: 10.1186/s12985-024-02421-y (PMC11218190; doi:10.1186/s12985-024-02421-y)
Supplement: Supplementary file 1 — Supplementary Material 1. [file 12985_2024_2421_MOESM1_ESM.docx]

Supplementary Table

| **Table 1: Graft versus Host Disease Prophylaxis and Frequency** | | | | |
| --- | --- | --- | --- | --- |
| **Variable** | **Malignant Hematological Disease**  **(MHD)**  **Group 1**  **N= 160** | **Non-malignant Hematological Diseases**  **(NHD)**  **Group 2**  **N= 199** | **Total**  **N = 359** | ***p* value** |
| Received prophylaxis for GvHD, N (%) | 152 (95) | 196 (98.5) | 348 (96.9) | 0.07 |
| Received calcineurin inhibitor, N (%)  None  Tacrolimus  Cyclosporin  Sirolimus | 8 (5)  35 (21.9)  117 (73.1)  0 | 3 (1.5)  6 (3)  22 (11.1(  168 (84.4) | 11 (3.1(  41 (11.4(  139 (38.7)  168 (46.8) | 0.001 |
| Received T-cell depletion, N (%)  Alemtuzumab  ATG | 0  44 (27.5( | 4 (2)  26 (13.1) | 4 (1.1(  70 (19.5) | 0.001 |
| Received methotrexate, N (%) | 79 (49.4) | 10 (5) | 89 (24.8) | 0.001 |
| Received mycophenolate mofetil, N (%) | 40 (25) | 21 (10.6) | 61 (17) | 0.001 |
| Received post-cyclophosphamide, N (%) | 17 (10.6) | 10 (5) | 27 (7.5) | 0.05 |
| Received steroids, N (%) | 63 (39.4) | 30 (15.1) | 93 (25.9) | 0.001 |
| Acute GvHD, N (%) | 54 (33.8) | 21 (10.6) | 75 (20.9) | 0.001 |
| Grades of acute GvHD, N (%)**^*^**  Grade I  Grade II  Grade III  Grade IV | 16 (29.6)  11 (20.4)  3 (5.6)  16 (29.6) | 12 (57)  5 (23.8)  2 (9.5)  1 (4.8) | 28 (37.3)  16 (21.3)  5 (6.7)  17 (22.7) | 0.04 |
| Types of acute GvHD, N  Oral/mucosa  Skin  Gut  Eye  Lungs  Others (e.g., liver) | 38  25  21  18  19  5 | 12  10  9  12  2  2 | 50 (66.7)  35 (46.7)  30 (40)  30 (40)  21 (28)  7 (9.3) | 0.07 |
| Chronic GvHD, N (%) | 26 (16.3) | 4 (2) | 30 (8.4) | 0.001 |
| Types of chronic GvHD, N  Skin  Oral  Gut  Eye  Others (liver, lungs) | 17  6  14  7  4 | 1  1  1  0  0 | 18 (60)  7 (23.3)  15 (50)  7 (23.3)  4 (13.3) | 0.05 |
| ^*^Data are missing for 8 patients | | | | |
